# Median Meld at Transplant Minus 3 Reduces the Mortality of Non-Hepatocellular Carcinoma Patients on the Liver Transplant Waitlist

**DOI:** 10.3390/curroncol31110519

**Published:** 2024-11-11

**Authors:** Panthea Pouramin, Susan E. Allen, Joseph L. Silburt, Boris L. Gala-Lopez

**Affiliations:** 1 Faculty of Medicine, Dalhousie University, Halifax, NS B3H 4R2, Canada; ponthea.pouramin@dal.ca (P.P.); joseph.silburt@nshealth.ca (J.L.S.); 2 Multi-Organ Transplant Program, Department of Surgery, Dalhousie University, Halifax, NS B3H 4R2, Canada; susane.allen@nshealth.ca; 3 Beatrice Hunter Cancer Research Institute, Halifax, NS B3H 0A2, Canada; 4 QEII Health Science Centre, Dalhousie University, 6-300 Victoria Bldg, 1276 South Park Street, Halifax, NS B3H 2Y9, Canada

**Keywords:** liver transplant, hepatocellular carcinoma, liver allocation, waitlist mortality, MELD score, MMaT-3

## Abstract

Liver transplants (LTs) are prioritized by mortality risk, which is estimated by MELD scores. Since hepatocellular carcinoma (HCC) patients present with lower MELD scores, they are allocated MELD exception points. Concerns persist that HCC recipients are over-prioritized, resulting in disproportionate waitlist mortality among non-HCC patients. We assessed whether the Median Meld at Transplant minus 3 (MMaT-3) scoring system would balance waitlist mortality and transplantation rates between HCC and non-HCC patients. We reviewed 266 patient charts listed for an LT from 2015 to 2023; 46.2% were listed in the MMaT-3 era. Amongst non-HCC patients, MMaT-3 implementation significantly increased 1-year transplant rate and reduced 1-year waitlist mortality among non-HCC patients (*p* = 0.003). Pre-MMaT-3 gaps in transplantation (*p* = 0.004) and waitlist dropout (*p* = 0.01) were eliminated post-implementation (*p* > 0.05). Amongst HCC patients, MMaT-3 implementation had no impact on the 1-year transplant rate (*p* = 0.92) or 1-year waitlist mortality (*p* = 0.66). Fine-gray proportional hazard multivariable analysis revealed that MMaT-3 significantly reduced waitlist mortality among non-HCC patients (asHR: 0.44, 95% CI [0.23, 0.83], *p* = 0.01) and limited impact on HCC patients (*p* = 0.31). MMaT-3 allocation did not significantly alter 2-year post-transplant survival for both populations. We show that the MMaT-3 system decreased the waitlist mortality of non-HCC patients with limited impacts on outcomes for HCC patients listed for an LT.

## 1. Introduction

Liver transplants (LTs) remain an essential yet limited resource for the treatment of liver failure and hepatocellular carcinoma alike. Transplantation remains the preeminent curative treatment option for HCC, a cancer that has an estimated survival rate of only 18% without curative interventions. HCC as an indication for liver transplant has increased in Canada from 2.3% of listed patients in 2000 to 32.4% in 2018. HCC has also consistently been the most common indication for liver transplant since that time [1]. At the same time, transplantation remains an essential curative treatment option for non-cancerous end-stage liver patients, who have a similarly high mortality rate (29.1%) when not transplanted [2].

The Model for End-Stage Liver Disease (MELD) score was developed to allocate livers most effectively, identifying patients with greater mortality risk and clinical urgency [3]. While the MELD score has brought consistency to patient listing priority, it does not adequately capture disease progression and mortality risk for HCC patients, who typically present with lower natural scores. To remedy this, HCC patients have traditionally been afforded “exception points” that artificially boost their MELD score and better compete with non-HCC patients for LTs [4,5,6,7,8,9].

Recent evidence suggests that current formulas for allocating exception points to HCC patients have overly preferred them, leading to inequitably worse outcomes for non-HCC patients [4,7,10,11,12]. To resolve this, the United Network for Organ Sharing (UNOS) developed the MELD score at Transplant minus three (MMaT-3) exception point system, a new allocation system that adjusts exception points based on a reflective calculation of local liver transplantation statistics [13,14]. Based on simulation studies, it was proposed that the MMaT-3 system would effectively balance waitlist outcomes between non-HCC and HCC patients and, in doing so, yield less overall mortality [15]. Nevertheless, the efficacy of the MMaT-3 system in real-world practice remains an open question [12,16], and more data are needed to interrogate its effectiveness.

Based on the inequity of existing HCC exception point systems in the literature [4,7,10,11,12] and our internal analysis, our institution adopted the MMaT-3 allocation algorithm. Here, we aimed to evaluate changes in LT waitlist mortality amongst HCC and non-HCC patients before and after the implementation of MMaT-3 to determine whether the MMaT-3 system more equitably distributes liver transplantations.

## 2. Material and Methods

### 2.1. Ethics

All research was conducted in accordance with both the Declarations of Helsinki and Istanbul. The project was approved by the Nova Scotia Health Research Ethics Committee (protocol#1027859). Given the retrospective nature of this study, the need for expressed written consent was waived.

### 2.2. Study Design and Patient Population

Reviewed in [9], in general, patients in Canada are prioritized based on the principle of “sickest first” as typically quantified by the MELD-Na score for adults. They are further categorized based on the CanWAIT system per their diagnosis and ambulatory status (e.g., intensive care unit) [16]. Typically, organs are offered provincially first and nationally if a recipient is not identified. However, a few exceptions exist to this rule, including acute liver failure, where patients can be listed nationally [16]. Exceptional MELD point allocation for HCC recipients differs from program to program, with some assigning a set number for all comers with a periodical increase, while other centers always list HCC patients with a predefined MELD value [9].

**Figure 1:**
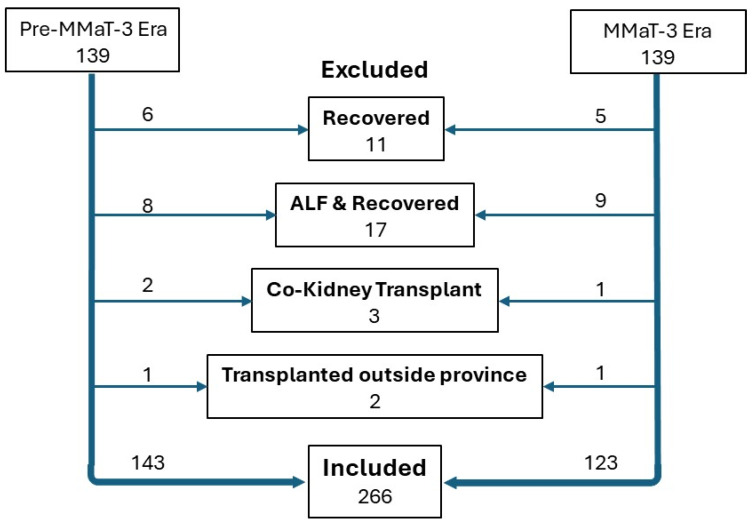
Flow chart of exclusions for the current study analysis.

This single-center retrospective analysis included patients from all four Atlantic Canada provinces who were 18 years and older and listed in the Multi-Organ Transplant Program (MOTP) database from 2015 to 2023 for a primary LT. Patients included during the 2015–2020 era received 22 MELD exception points at the time of listing, whereas subjects included in the 2020–2023 era received MELD exception points according to the calculated MMaT-3. The study divided the patient population into two periods: Group 1: Pre-MMaT-3 era (1 March 2015 to 1 March 2020); and Group 2: the MMaT-3 era (1 March 2020 until 16 April 2024). The MMaT-3 exception point calculation was initiated and calculated annually on 1 January based on the previous year’s MELD score of transplanted patients. Also, a 3-month wait period before assigning exception points started on 1 March 2020. If the patient’s natural MELD was above the MMaT-3 exception points at any time, the natural MELD was used for listing purposes. Subjects were excluded from this study if they received an LT for an acute liver failure, if they received multi-organ transplants, if they were withdrawn from the list because their clinical status improved, if they were not transplanted out of our center, or if they were re-transplanted within 30 days. A flow chart of exclusions is shown in Figure 1. For all analyses, we calculated the MELD-Na (Scientific Registry of Transplant Recipients) [17], which is referred to throughout the manuscript as the “MELD score”.

### 2.3. Outcomes

Primary outcomes included waitlist dropout and transplantation. Once a patient is listed for an LT, they may “withdraw” or drop off the list for 1 of 3 reasons: mortality, negative alterations in their clinical condition making them ineligible for a liver transplant, or improvement in clinical condition to the point where the liver transplant would not be necessary (excluded from this analysis). Secondary outcomes included 2-year post-transplant mortality and 2-year HCC recurrence. The Risk Estimation of Tumor Recurrence After Transplant (RETREAT) score was calculated post-transplant in HCC patients to evaluate the potential impact of the two allocation policies.

### 2.4. Statistics

Pre- and post-MMaT3 implementation were assessed in non-HCC and HCC patients separately. In univariate analysis, categorical variables were assessed via chi-square analysis, and continuous variables were assessed via the Mann–Whitney U test. Multivariable waitlist dropout rates were evaluated using fine-gray proportional hazard regression models which better account for the competing endpoint events (i.e., transplantation or dropout) by producing adjusted sub-distribution hazard ratios (asHRs) [14]. In parallel, we conducted a multivariable difference-in-difference analysis via a linear probability model [18] to evaluate the change in probability for non-HCC patients to drop out and be transplanted as a result of MMaT-3 implementation. For both multivariable analyses, we a priori included natural MELD, age, and BMI, which have previously been shown to be significantly associated with waitlist dropout [19,20]. Descriptive and univariate analyses were performed in SPSS (version 28, IBM, Armonk, NY, USA). Fine-gray analysis was conducted using the Cmprsk package (version 1.1.1; available from https://pypi.org/project/cmprsk/, accessed on 3 October 2024), and difference-in-difference analysis was conducted using the statsmodel [21] in Python (version 3.8). 

## 3. Results

### 3.1. Patient Characteristics

This study included 266 patients, with 181 non-HCC and 85 HCC subjects. In total, 123 (46.2%) participants were treated in the MMAT-3 allocation era. Demographically, both non-HCC and HCC patients were predominately of male sex (63.0% vs. 69.4%, *p* = 0.30) and of a BMI outside the normal range (28.4 IQR: [24.4, 32.8] vs. 29.0 [25.9, 33.7], *p* = 0.16). However, compared to non-HCC patients, HCC patients were older (53 IQR: [46, 60] vs. 63 IQR: [58, 65], *p* < 0.001) (Table 1). 

### 3.2. Impacts on Non-HCC Patients

We asked whether the switch to the MMaT-3 exception point system balanced the waitlist dropout rate for non-HCC patients (Table 2). In the pre-MMaT-3 era, non-HCC patients experienced significantly higher 1-year dropout (non-HCC: 27.1% vs. HCC: 8.5%, *p* = 0.01) and lower 1-year transplantation rates compared to HCC patients (non-HCC: 56.3% vs. HCC: 80.9%, *p* < 0.01). Following the implementation of MMaT-3, non-HCC patients experienced a significant increase in 1-year transplant rates (pre: 56.3% vs. post: 73.4%, *p* = 0.03) as well as a significant reduction in the 1-year dropout rate (pre: 27.1% vs. post: 12.5%, *p* = 0.03). As such, within the MMaT-3 implementation era, non-HCC and HCC patients showed a statistically indistinguishable 1-year dropout (non-HCC: 12.5% vs. HCC: 11.4%, *p* = 0.88) and 1-year transplantation rates (non-HCC: 73.4% vs. HCC: 80.0%, *p* = 0.47). Upon univariate analysis, the time to transplantation was not significantly different (pre: 93 days IQR [36, 220] vs. post: 76 days [15, 178], *p* = 0.18). Amongst non-HCC patients who were transplanted, there was no difference in 2-year post-transplant mortality pre- and post-MMaT-3 (pre: 13.8% vs. post: 15.8%, *p* = 0.787).

### 3.3. Impacts on HCC Patients

Among HCC patients (Table 2), there was no significant change in 1-year transplant rate (pre: 80.9% vs. post: 80.0%, *p* = 0.92), 1-year dropout rate (pre: 8.5% vs. post: 11.4%, *p* = 0.66), or time to transplantation (pre: 134 days IQR [53, 282] vs. post: 150 days IQR [81, 223], *p* = 0.91). Moreover, 2-year post-transplantation mortality did not differ between pre- and post-MMaT3 eras (pre: 20.3% vs. post: 6.3%, *p* = 0.182). Similarly, 2-year HCC recurrence (pre: 7.0% vs. post: 12.5%, *p* = 0.498), as well as transplant-time RETREAT scores, a prognostic score of HCC recurrence, did not differ between pre- and post-MMaT3 eras (pre: 4 [2, 4] vs. post: 4 [2, 5], *p* = 0.89).

### 3.4. Predictors of Waitlist Dropout

In multivariable fine-gray analysis (Table 3), after adjusting for age, BMI, and natural MELD score, adopting the MMaT-3 program significantly reduced the incidence of waitlist dropout among non-HCC patients (asHR: 0.44, 95% CI [0.23, 0.83]; *p* = 0.01) (Table 3). This analysis further identified the natural MELD score (asHR: 1.09, 95% CI [1.04, 1.13]; *p* < 0.001) and age (asHR: 1.05, 95% CI [1.01, 1.08]; *p* < 0.01) as significant predictors of waitlist dropout among non-HCC patients. By contrast, among HCC patients, implementation of the MMaT-3 program did not significantly affect waitlist dropout rates (asHR: 1.74, 95% CI [0.51, 6.01]; *p* = 0.31). Also, neither age (*p* = 0.29) nor BMI (*p* = 0.35) impacted waitlist dropout. However, there was a trending association with increasing MELD score (asHR: 1.11 95% CI [0.99, 1.25], *p* = 0.07).

In parallel, we conducted a multivariable difference-in-difference analysis (Table 4). Following MMaT-3 implementation, relative to HCC patients, non-HCC patients saw a trending reduction in 1-year dropout (−18.6% 95% CI (−37.8%, 0.5%), *p* = 0.057) and a trending increase in 1-year transplantation (23.0%, 95% CI (−0.6%, 46.7%), *p* = 0.056). Similar to the fine-gray analysis, natural MELD (*p* < 0.0001) and age (*p* = 0.02) were significant predictors of dropout; however, they were not significantly associated with a change in transplantation rates.

## 4. Discussion

This study reflects on the impact of the first implementation of the MMaT-3 liver allocation policy in a Canadian center. Here, we asked whether incorporating the MMaT-3 allocation algorithm would more equitably allocate liver transplants to non-HCC patients and reduce their waitlist dropout. Through multivariable fine-gray analysis, we found that the MMaT-3 program reduced the risk of waitlist dropout for non-HCC by over half. Moreover, the apparent discrepancy in transplantation and dropout rates between non-HCC and HCC patients from the pre-MMaT-3 era were statistically equalized in the post-MMaT-3 era, with 7 in 10 non-HCC patients and 8 in 10 HCC patients receiving a transplant. Conversely, implementing the MMaT3 program had no discernable impact on HCC patient dropout and transplantation rates, which were statistically indistinguishable from the pre-MMaT3 era. Altogether, we report a trend that following MMaT-3 implementation, relative to HCC patients, non-HCC patients were 18.6% less likely to drop out and 23.0% more likely to be transplanted than before.

Adopting the MMaT-3 system remains a crucial unresolved debate in the transplantation community. Those who favor adoption point to the fact that HCC patients are more stable while waiting for liver transplants than non-HCC patients [4,12]. In an analysis of three policy eras, reducing exception points for HCC patients while reducing total transplantations for HCC participants had no statistically significant impact on waitlist dropout [22]. Advances in pre-transplant HCC treatment have substantially improved outcomes for this population. For example, following a 2-year follow-up, Mehta et al. found that low-risk patients who responded well to locoregional therapy had a 2-year dropout of only 1.6% [23]. Under the pre-MMaT-3 era, low-risk patients would likely be prioritized more than their higher mortality non-HCC counterparts [4,12].

The argument is, therefore, that reprioritizing liver access from HCC patients to non-HCC patients through limiting HCC exception points will reduce non-HCC dropout rates while having a limited impact on HCC dropout. In support of this hypothesis, a large retrospective cohort analysis of the UNOS transplant sharing service found that MMaT-3 adoption reduced waitlist dropout rates for non-HCC recipients and equalized the risk for dropping out with HCC patients [24]. Multivariable regression analysis showed no association between MMaT-3 implementation and dropout amongst HCC patients. However, a subgroup analysis showed that in long wait time regions, implementation of MMaT-3 did increase the risk for dropout amongst HCC patients [24]. A previous study of the UNOS dataset similarly found that MMaT-3 achieved liver allocation and dropout parity between HCC and non-HCC patients [14]. This analysis further showed that while dropout rates among HCC patients remained stable between pre- and post-MMaT-3 eras, 1-year transplantation rates for HCC patients were reduced to a rate equal to that of non-HCC patients [24]. These results are consistent with our work and point to the MMaT-3 allocation system as successful in equalizing outcomes between HCC and non-HCC patients.

Nevertheless, while we and others suggest that MMaT-3 has minimal impact on waitlist dropout for HCC patients, the effect of MMaT-3 on overall survival remains unclear. Proponents of MMaT-3 point to locoregional therapy (LRT) as a vital bridging therapy [25] that, for some populations, can safely delay transplantation [12]. However, this point remains controversial, as a recent meta-analysis evaluating the efficacy of LRT in within-Milan criteria patients found limited improvements in dropout rates and overall survival [26]. On the other hand, a meta-analysis of liver transplantation delays found that prolonging transplantation wait times for HCC patients beyond 90 days may itself have a limited impact on overall survival [27], therefore suggesting that HCC patients are relatively stable on the waitlist.

Our work shows that MMaT3 allocation did not significantly impact 2-year post-transplantation mortality rates for either non-HCC or HCC patients. Similarly, amongst HCC patients, 2-year HCC recurrence, as well as RETREAT scores, an indicator of HCC recurrence, did not change in the MMaT-3 era. This suggests that minor delays in liver transplantation for HCC patients do not precipitate significant increases in the risk for HCC recurrence. Given that our study is underpowered to evaluate small effects on HCC recurrence and survival, we should not overinterpret these results, and it is possible that increased recurrence will manifest at later time points. Consistent with our work, a recent study of the UNOS database found that switching to MMaT3 allocation was associated with a small increased risk of all-cause mortality for HCC patients [13]. Notably, this increased mortality was not attributable to HCC recurrence but instead was primarily driven by an increase in infection-related deaths. Moreover, similar to our work, the MMaT-3 era was not associated with the pathological progression of HCC compared to the pre-MMaT-3 era. Ultimately, any such slight increase in mortality amongst HCC patients must be weighed against the benefit experienced by non-HCC patients as a result of having greater access to liver transplantation, which, based on our and others’ analysis, is significant.

Our study has several limitations. While we detected no statistically significant decrease in dropout among HCC patients, we must recognize our relatively low sample size and event rate from which to draw comparisons. Thus, we cannot rule out that our analysis was underpowered to measure a small negative impact on HCC patients. Moreover, we cannot rule out that the impacts of the MMaT-3 policy will manifest greater HCC recurrence and mortality beyond the 2 years evaluated in our patient cohort. Thus, continued follow-up will be necessary to evaluate the impact of the MMaT-3 policy on HCC patients and help to clarify whether HCC patients are impacted in the longer term.

## 5. Conclusions

In conclusion, we show that MMaT3 reduced waitlist dropout rates among non-HCC patients while having a minimal impact on HCC waitlist dropout rates. In doing so, the MMaT-3 allocation policy eliminated a previous discrepancy in transplantation and dropout outcomes between non-HCC and HCC patients. Thus, concerning waitlist dropout rates in our population, the MMaT3 program achieved its aim of more equitably distributing liver transplantations.

## Figures and Tables

**Table 1:** Patients’ clinical and demographic factors disaggregated by hepatocellular carcinoma status.

	non-HCC (*n* = 181)	HCC (*n* = 85)	*p*-Value
Post-MMaT-3 (percentage)	85 (47.0%)	38 (44.7%)	0.731
Male [count (percentage)]	114 (63.0%)	59 (69.4%)	0.305
Age (years)	53 (46, 60)	63 (58, 65)	<0.001
BMI (kg/m^2^)	28.4 (24.4, 32.8)	29.0 (25.9, 33.7)	0.157
Primary Liver Pathology			
NASH	47 (26.0%)	-	-
Alcohol-related liver disease	43 (23.8%)	-	-
Bile duct disease *	48 (26.5%)	-	-
Other **	43 (23.8%)	-	-

* Primary sclerosing cholangitis, secondary sclerosing cholangitis, and primary biliary cirrhosis. ** HCV, autoimmune hepatitis, cryptogenic liver cirrhosis, hemochromatosis, polycystic liver disease, and others. HCC: hepatocellular carcinoma; BMI: body mass index; NASH: nonalcoholic steatohepatitis.

**Table 2:** Clinical outcomes disaggregated by transplantation allocation policy and hepatocellular carcinoma status.

	Pre-MMaT-3	Post-MMaT-3	Pre vs. Post *p*-Value
1-year transplant [count (percentage)]			
non-HCC	54 (56.3%)	47 (73.4%)	0.027
HCC	38 (80.9%)	28 (80.0%)	0.923
HCC vs. non-HCC *p*-value	0.004	0.466	
1-year dropout [count (percentage)]			
non-HCC	26 (27.1%)	8 (12.5%)	0.027
HCC	4 (8.5%)	4 (11.4%)	0.66
HCC vs. non-HCC *p*-value	0.010	0.876	
2-year post-transplant mortality [count (percentage)]			
Non-HCC	9 (13.8%)	6 (15.8%)	0.787
HCC	9 (20.9%)	1 (6.3%)	0.182
HCC vs. non-HCC *p*-value	0.334	0.341	
2-year HCC recurrence [count (percentage)]	3 (7.0%)	2 (12.5%)	0.498
MELD natural [median (IQR)]			
non-HCC	21 (15, 25)	20 (14, 27)	0.480
HCC	10 (8, 12)	11.50 (8, 14.25)	0.153
HCC vs. non-HCC *p*-value	<0.0001	<0.0001	
Time to dropout [median (IQR)]			
non-HCC	91 (25, 251)	29 (5, 69)	0.079
HCC	118 (33, 249)	56 (33, 198)	0.762
HCC vs. non-HCC *p*-value	0.940	0.179	
Time to transplant [median (IQR)]			
non-HCC	93 (36, 220)	76 (15, 178)	0.180
HCC	134 (53, 282)	150 (81, 223)	0.906
HCC vs. non-HCC *p*-value	0.438	0.094	
RETREAT score at transplant [median (IQR)]	4 (2, 5)	4 (2, 4)	0.886

HCC: hepatocellular carcinoma; MMaT-3: Median Meld at Transplant minus 3; IQR: interquartile range; RETREAT: Risk Estimation of Tumor Recurrence After Transplant.

**Table 3:** A multivariable fine-gray proportional hazard regression analysis of waitlist dropout.

	Non-HCC				HCC			
	sHR (95% CI)	*p*-Value	asHR (95% CI)	*p*-Value	sHR (95% CI)	*p*-Value	asHR (95% CI)	*p*-Value
Age (years)	1.04 (1.00, 1.06)	0.024	1.05 (1.01, 1.08)	0.006	1.07 (0.92, 1.24)	0.350	1.08 (0.94, 1.23)	0.287
BMI (kg/m^2^)	1.03 (0.98, 1.08)	0.296	1.04 (0.98, 1.09)	0.191	1.04 (0.935)	0.433	1.06 (0.94, 1.19)	0.354
MELD Natural	1.07 (1.03, 1.11)	0.0002	1.09 (1.04, 1.13)	<0.0001	1.18 (1.00, 1.24)	0.040	1.11 (0.99, 1.25)	0.072
Post-MMaT-3	0.490 (0.26, 0.93)	0.028	0.44 (0.23, 0.83)	0.011	1.94 (0.56, 6.7)	0.298	1.74 (0.51, 6.01)	0.379

asHR: adjusted subhazard ratio; sHR: subhazard ratio; HCC: hepatocellular carcinoma; MMaT-3: Median Meld at Transplant minus 3; BMI: body mass index; MELD: Model for End-Stage Liver Disease.

**Table 4:** A multivariable difference-in-difference linear probability model of 1-year waitlist dropout and 1-year transplantation.

	1-Year Dropout				1-Year Transplantation			
	Unadjusted Probability Difference (95% CI)	*p*-Value	Adjusted Probability Difference (95% CI)	*p*-Value	Unadjusted Probability Difference (95% CI)	*p*-Value	Adjusted Probability Difference (95% CI)	*p*-Value
Non-HCC vs. HCC	18.6% (5.1%, 19.5%)	0.007	9.0% (−6.0%, 24.1%)	0.239	−24.6% (−40.5%, −8.7%)	0.003	−24.6% (−43.3%, −0.06%)	0.01
Post- vs. Pre-MMaT3	7.3% (−9.2%, 23.8%)	0.386	5.8% (−10%, 21.5%)	0.471	−4.5% (−24.1%, 15.0%)	0.648	−4.7% (−24.1%, 14.9%)	0.639
Non-HCC Post-MMaT3	−19.1% (−39.1%, 0.09%)	0.061	−18.6% (−37.8%, 0.5%)	0.057	22.4% (−1.2%, 46.0%)	0.063	23.0% (−0.6%, 46.7%)	0.056
Age (years)	-	-	0.6% (0.1%, 1.1%)	0.020			−0.4% (−1.0%, 0.2%)	0.187
BMI (kg/m^2^)	-	-	0.5% (−0.3%, 1.3%)	0.203			−0.5% (−1.4%, 0.3%)	0.173
MELD Natural	-	-	1.4% (0.8%, 2.0%)	<0.0001			−0.3% (−1.1%, 0.3%)	0.295

HCC: hepatocellular carcinoma; MMaT-3: Median Meld at Transplant minus 3; BMI: body mass index; MELD: Model for End-Stage Liver Disease.

## Data Availability

The data presented in this study are available on request from the corresponding author due to privacy and ethical restrictions.

## List of Abbreviations

BMI	body mass index
HCC	hepatocellular carcinoma
IQR	interquartile range
MELD	Model for End-Stage Liver Disease
MMaT-3	Median Meld at Transplant minus 3
MOTP	Multi-Organ Transplant Program
NASH	nonalcoholic steatohepatitis
Non-HCC	non-hepatocellular carcinoma
LRT	locoregional therapy
LT	liver transplant
RETREAT	Risk Estimation of Tumor Recurrence After Transplant
UNOS	United Network for Organ Sharing
sHR	subhazard ratio
